# Changing the biological Rosetta stone: the (commercial) potential of recoded microbes

**DOI:** 10.1111/1751-7915.13466

**Published:** 2019-08-01

**Authors:** Javier Santos‐Moreno, Yolanda Schaerli

**Affiliations:** ^1^ Department of Fundamental Microbiology University of Lausanne Biophore Building 1015 Lausanne Switzerland

In 1822, the French Egyptologist Jean‐François Champollion announced the deciphering of the Rosetta stone, the key for translating Egyptian hieroglyphs, thereby enabling an unprecedented insight into ancient Egypt history. Life has its own Rosetta stone, the genetic code, decoded by Marshall Nirenberg in 1960s. While the genetic code is universal (albeit exceptions) and has not undergone major changes since the last universal common ancestor, technological progress allows synthetic biologists to increasingly re‐write the biological Rosetta.

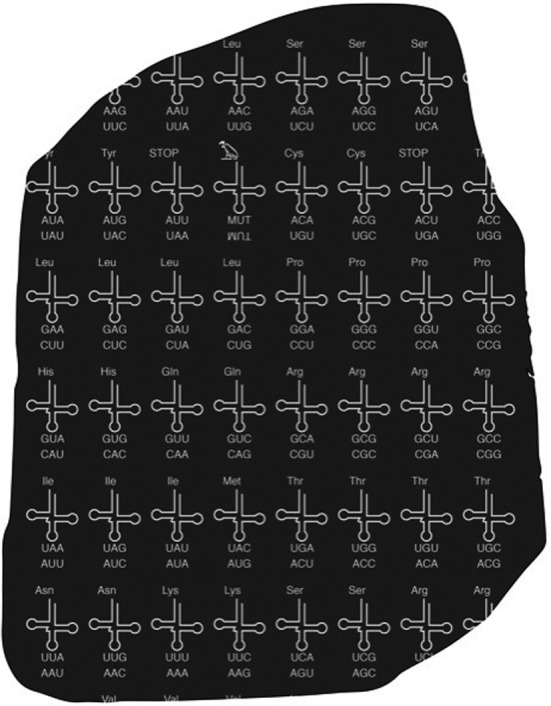

Researchers can set new rules at several levels of life's language. First, the language can be modified at its most basic structure, the *alphabet*. While DNA uses only four letters (nucleotides) to encode messages, an extension to the alphabet can be envisioned. For instance, a recent study reported an extended, 8‐letter (‘hachimoji’) DNA and RNA, in which the natural nucleotides coexist with four synthetic building blocks (Hoshika *et al*., 2019). While 4 letters can encode 64 (4^3^) triple codons, 8 letters can be combined to 4096 (8^3^) codons, thus massively increasing the information that can be encoded. So far, the 8‐letter code has only been transcribed (into RNA) but not read, that is translated into a polypeptide sequence. However, new codons generated by a 6‐letter code (6^3^ = 216) have already been used to incorporate non‐natural amino acids (nnAAs) into proteins (Zhang *et al*., 2017).

Researchers can also actuate on how groups of letters (*words*) encode a meaning. In nature, the 64 codons code for 20 amino acids plus the translation termination signal. Degeneracy is a major characteristic of the genetic code and has diverse and important roles. At the same time, it leaves some room for recoding: the redundant codon number can be reduced by obviating the use of a given codon in favour of a synonymous one, thus ‘freeing’ the former, which can be reassigned to a nnAA.

Codon reassignment to a nnAA requires an orthogonal translation system: the charging of the corresponding tRNA exclusively with the reassigned amino acid by an aminoacyl‐tRNA synthetase that does not interact with any of the other tRNAs (Chin, 2014). The most common codon to be reassigned is the amber stop codon TAG, which is the least abundant codon across the genome. However, competition between the release factor 1 (RF1) and the orthogonal tRNA results in ambiguous and inefficient translation that produces variable proteins. Moreover, uncontrolled incorporation of nnAAs into the genome reduces cellular fitness. The genome‐wide re‐writing of the reassigned codon (TAG) as a synonymous codon (e.g. TAA) and the deletion of RF1 solves this problem. In 2011, synthetic biologists generated an *Escherichia coli* strain that has all 321 TAG stop codons replaced with TAA (Isaacs *et al*., 2011). This approach has been extended to replace other synonymous codons (Isaacs *et al*., 2011; Ostrov *et al*., 2016; Fredens *et al*., 2019). To date, the largest recoded genome is that of *E. coli* Syn61 recently created by the Jason Chin labarotary, in which > 18k codons were recoded to yield a 61‐codon genome (Fredens *et al*., 2019).

But this record will probably not last long. The collaborative synthetic yeast genome project is already well advanced in the endeavour of creating the first synthetic eukaryotic genome, *Saccharomyces cerevisiae* Sc2.0. Among other new design features, it will have all TAG stop codons recoded to TAA (Richardson *et al*., 2017). Similarly, the synthesis of a recoded human genome is sought by an international consortium (Boeke *et al*., 2016). Inspired by the Human Genome Project (HGP‐read), which in 2004 yielded the sequence of the human genome with the concomitant improvement of DNA sequencing technology, cost and quality, the HGP‐write aims to engineer large genomes and thus contribute to reduce costs of design and testing of synthetic genomes.

Indeed, genome recoding is very much dependent on the development of enabling technologies. Some genome recoding projects may require a relatively low number of changes to be made, and thus, editing the existing genome may reveal a realistic and even a convenient option (Isaacs *et al*., 2011). However, where large‐scale modifications are desired, rebuilding the genome from chemically synthesized DNA might be more efficient. The assembly of the synthetic genome can either be carried out sequentially, by replacing segments in a pre‐defined order (Fredens *et al*., 2019), or simultaneously, by assembling the artificial genome *de novo* in a single reaction from smaller constituent parts (Hutchison *et al*., 2016). We anticipate that decreasing costs of DNA synthesis will boost such genome‐wide recoding endeavours in the mid‐term.

But, talking business, where do all these recoding efforts lead to? Can the recoding of nature's language also set new rules in the life science market? Well, we think it can. Genome recoding holds a huge potential not only for academic research, but also for commercial applications. First, the use of nnAAs offers great promise for the development of new catalysts of industrial interest. The 20 natural amino acids limit the number of reactions that enzymes can perform. NnAAs expand the repertoire and provide new properties that can result in novel functions never found before in nature (Agostini *et al*., 2017). As the recent Nobel Prize laureate Frances Arnold has shown, enzyme catalysts can outperform chemical ones, and thus, nnAAs can potentially yield novel *super*catalysts to replace conventional chemical synthesis, which can use a lot of energy and toxic chemicals, with more environmentally friendly processes.

Second, therapeutic protein production can also greatly benefit from the *à la carte* incorporation of nnAAs. Indeed, while currently most proteins of therapeutic relevance are produced in eukaryotic systems, a prokaryote‐based production would offer important advantages, such as easier genetic manipulation, faster and cheaper production, and reduced contamination rates. Yet, standard prokaryotic ‘workhorses’ like *E. coli* are unable of most eukaryotic post‐translational modifications (PTMs). Codon reassignment has been used to bypass the prokaryotic limitations and incorporate eukaryotic PTMs (Neumann *et al*., 2008; Nguyen *et al*., 2009; Virdee *et al*., 2011). Similarly, nnAAs can help improving protein functionalization, for example for producing bispecific antibodies or antibody–drug conjugates, or for attaching polyethylene glycol chains (PEG) to improve protein stability (Huang and Liu, 2018).

Third, chemical synthesis of genomes and codon reassignment is also a promising strategy to produce live‐attenuated vaccines. Viruses with genomes recoded with synonymous mutations have been reported to trigger a wild‐type immune response since the protein sequence is unaltered, but they have a reduced viability due to sub‐optimal replication or translation, resulting in safer vaccines (Wang *et al*., 2018).

Fourth, recoded genomes can provide increased virus resistance to their hosts, since a virus infecting a cell with re‐purposed tRNA(s) is unable to properly translate its genes (Lajoie *et al*., 2013). Virus resistance is a desirable property for (almost) any culture of (eukaryotic or prokaryotic) cells used in industrial production of goods such as therapeutic proteins, food or chemicals. For example, bacteriophage contamination is a relevant issue in industrial fermentations: in the dairy industry, which produced 817 million tons of milk in 2016, phage infection of the starter cultures is the main factor causing fermentation failures (Fernandez *et al*., 2017).

Finally, a major advantage of recoded organisms is their potential for biocontainment. On one hand, codon‐reassigned strains are virtually reluctant to horizontal gene transfer (both incoming and outcoming), since incoming DNA is unreadable in the engineered strain, and, similarly, synthetic genes are not properly translated in a natural host. Even more, this *passive* incompatibility can be further improved *via* an active mechanism: a non‐recoded toxin gene in the recoded organism would prevent the transfer of the artificial cassette to natural strains, while selecting against reacquiring the native tRNA machinery (Kuo *et al*., 2018). On the other hand, codon reassignment also allows to make the survival of the organism dependent on the presence of the nnAAs, notably through the insertion of nnAAs in essential genes (Mandell *et al*., 2015; Rovner *et al*., 2015).

As opposed to genetically modified organisms (GMOs), improved biocontainment of genetically recoded organisms (GROs) could have major scientific and economic implications. From a scientific perspective, it is obviously desirable to keep biocontainment to a maximum, unless a strain is purposely engineered for genetic exchange. From a socioeconomic view, a greater control over engineered strains might have a direct impact on public opinion and, in turn, on policy makers. Consumers still perceive GMOs as rather negative and receive GMO information mostly from non‐scientific sources (Wunderlich and Gatto, 2015). Ensuring a reliable biocontainment might serve to reassure the population and revisit restrictive regulations, which might have enormous economic implications for the agricultural and biotech industries. Ironically, while engineered organisms are still often seen as a sort of *Frankenstein*, making them *Frankenstein*er might actually make them safer and change the perception of society and stakeholders.

For half a century, reading the DNA has generated major benefits to humankind, both scientifically and economically. Now, the ability to write the DNA and to include changes such as the here discussed genome recoding, but also streamlining and stabilization, promises to bring a comparable revolution that will likely imply profound changes in research, health, agriculture and bio‐based industrial production.

## Conflict of interest

None declared.
